# Validation of hippocampal biomarkers of cumulative affective experience

**DOI:** 10.1016/j.neubiorev.2019.03.024

**Published:** 2019-06

**Authors:** Colline Poirier, Melissa Bateson, Fabio Gualtieri, Elena A. Armstrong, Grace C. Laws, Timothy Boswell, Tom V. Smulders

**Affiliations:** a Institute of Neuroscience, Newcastle University, UK; b School of Natural and Environmental Sciences, Newcastle University, UK; c Centre for Behaviour and Evolution, Newcastle University, UK

**Keywords:** Cumulative experience, Hippocampus, Neurogenesis, Affective states, Animal welfare, Plasticity, Biomarker, Validity, Stress, Quality of life

## Abstract

Progress in improving the welfare of captive animals has been hindered by a lack of objective indicators to assess the quality of lifetime experience, often called cumulative affective experience. Recent developments in stress biology and psychiatry have shed new light on the role of the mammalian hippocampus in affective processes. Here we review these findings and argue that structural hippocampal biomarkers demonstrate criterion, construct and content validity as indicators of cumulative affective experience in mammals. We also briefly review emerging findings in birds and fish, which have promising implications for applying the hippocampal approach to these taxa, but require further validation. We hope that this review will motivate welfare researchers and neuroscientists to explore the potential of hippocampal biomarkers of cumulative affective experience.

## Highlights

• Recent knowledge on hippocampal structural plasticity is reviewed.
• This knowledge is harnessed to develop biomarkers of cumulative experience.
• Hippocampal plasticity is shown to have construct, content and criterion validity in mammals.
• The biomarkers require further validation to be used in birds and fish.
• We discuss some practical considerations to implement the biomarkers.

## 1 Introduction

The general public have long been concerned with the welfare of laboratory, farm, zoo and companion animals. This concern stems from the assumption that, like humans, many other animals can consciously experience affective states. However, because the subjective conscious component of affective states described by humans as ‘feelings’ is difficult to assess in non-verbal animals, welfare researchers usually focus on measuring the physiological and behavioral components of affective states. Therefore, following Paul and colleagues (Paul et al., 2005), when we use the terms ‘affective states’ and ‘affective experience’, we refer only to objectively measureable physiological and behavioral responses. Furthermore, we adopt the common two-dimensional model of affect in assuming that core affective states (i.e. excitement, contentment, sadness and anxiety) can be described as combinations of the valence (pleasantness vs. unpleasantness) and intensity (arousal) of the animals’ experiences (Mendl et al., 2010).

Traditionally animal welfare science has been mostly concerned with the current short-term affective state of an individual resulting from a particular event (Boissy et al., 2007; Mendl et al., 2010). More recently, however, emphasis has shifted towards the lifetime experience of animals, reflected in the related concepts of ‘quality of life’ and ‘cumulative experience’, and this shift is now reflected in legislation regulating animal use in science (Directive 2010/63/EU) and recommendations for the farming industry (Farm Animal Welfare Council, 2009). Cumulative experience can be defined as the net impact of all the events that affect the welfare of an animal over its lifetime, be it negatively, positively, and/or by way of amelioration (definition adapted from Pickard, 2013). In order to avoid confusion with the non-affective definition of experience (e.g. learning and memory), we will henceforth refer to this as cumulative affective experience.

The shift in concern in the field of animal welfare research from acute to cumulative affective experience raises the question of how to measure the latter. For regulatory purposes, cumulative affective experience is currently assessed using crude objective physical indicators such as body weight, that lack sensitivity for detecting subtle changes in welfare, and clinical impression (used by veterinarians), which is subjective and open to disagreement. Other proposed methods also suffer from limitations (Bateson and Poirier, 2019). One potential solution might be to record all the putatively positive and negative stimuli that an animal has been exposed to over time and add these up to produce a measure of cumulative affective experience. However, different animals respond very differently to the same stimuli, and the Pickard report (on the assessment of cumulative affective experience in non-human primates used in neuroscience research) reached the conclusion that ‘there is no mathematical way of integrating all positive and negative events in an animal’s life (Pickard, 2013; Bateson, 2016). We therefore need an indicator that reflects each individual animal’s response to the stimuli to which it has been exposed, rather than a record of the stimuli themselves.

Here, we use recent evidence to argue that such a marker of cumulative affective experience can be found in the brain, and more specifically in the hippocampus, a well-studied brain area involved in learning, memory, and stress regulation. Following a general introduction to the mammalian hippocampus, we will explore the criterion, construct, and content validity of these hippocampal biomarkers as indicators of cumulative affective experience in mammals. We will then discuss confounding factors and propose potential strategies to control for them. Finally, we will present preliminary evidence supporting the potential of these hippocampal biomarkers for assessment of cumulative affective experience in non-mammalian species as well. We will conclude with some practical considerations for implementing these markers in various settings.

## 2 General introduction to the mammalian hippocampus

The mammalian hippocampal formation (henceforth hippocampus) is a bilateral, oblong, forebrain structure. The hippocampus can be subdivided into three anatomically distinct fields visible in cross section: the subiculum, the cornu ammonis and the dentate gyrus (Witter, 2009). Recent evidence drawn from gene expression, anatomical, and functional connectivity studies indicates that the hippocampus can be further subdivided into three main regions along its longitudinal axis (antero-posterior axis in primates, ventro-dorsal in rodents) (for a review, see Strange et al., 2014). Since the anterior hippocampus in primates is homologous to the ventral hippocampus in rodents, and the posterior hippocampus in primates is homologous to the dorsal hippocampus of rodents, we will henceforth refer to these subdivisions as anterior/ventral and posterior/dorsal respectively.

While the hippocampus is perhaps better known for its role in learning and memory processes, it also plays a central role in emotional regulation (Fanselow and Dong, 2010; Moser and Moser, 1998). One way the hippocampus regulates acute affective experiences is by applying strong negative feedback to the hypothalamic-pituitary-adrenal (HPA) axis, a central component of the stress response system (de Kloet et al., 1998; Jacobson and Sapolsky, 1991). The activation of the HPA axis by a stressor induces the release of glucocorticoid hormones (corticosterone in rodents, cortisol in other mammals) into the circulating blood. Following termination of the stressor, glucocorticoid concentrations slowly decrease to pre-stress levels and this recovery is regulated by negative feedback of glucocorticoids onto their receptors in the brain, especially in the hippocampus. The hippocampus therefore exerts regulatory control over the HPA axis (for recent reviews, see de Kloet et al., 2016; Suri and Vaidya, 2015).

Behavioral studies indicate that the two main functions of the hippocampus, learning and memory, and emotional regulation, are spatially segregated (though the segregation is not perfect), with the posterior/dorsal part of the hippocampus mainly involved in learning and memory, and the anterior/ventral part mainly involved in affective experiences. Evidence supporting this spatial segregation comes from studies showing that lesions in the ventral hippocampus of rats impair defensive fear expression but not spatial memory, while lesions in the dorsal part have the opposite effect (for reviews see Bannerman et al., 2004; Fanselow and Dong, 2010; Moser and Moser, 1998; Strange et al., 2014). This functional segregation is supported by different anatomical connectivity, with the anterior/ventral hippocampus being mainly connected to brain regions involved in emotional regulation and the posterior/dorsal region being connected to brain regions involved in spatial memory (for a review see Strange et al., 2014).

The hippocampus, and more specifically the dentate gyrus, is one of only a few brain regions where neurogenesis, the birth of new neurons, occurs throughout postnatal life in the healthy mammalian brain (Ming and Song, 2011). The rate of neurogenesis varies between species and the existence of adult neurogenesis has been questioned in some mammals, especially humans (Sorrells et al., 2018). However, the dominant opinion is that the claim that neurogenesis occurs in adult humans at a functionally-relevant rate is robust (Boldrini et al., 2018; Kempermann et al., 2018; Moreno-Jiménez et al., 2019) (for a similar debate on the existence of adult neurogenesis in bats with a similar positive conclusion, see Chawana et al., 2014). New neurons are born in the dentate gyrus where they mature and become functional, growing axons that connect to other hippocampal subdivisions. An increasing number of studies suggests that neurogenesis plays an important role in learning and memory and also in emotional regulation (for reviews see Fanselow and Dong, 2010; Moser and Moser, 1998; Strange et al., 2014). Furthermore, the spatial segregation of learning and memory vs. affective experiences observed at the level of the whole hippocampus is suspected to be mirrored by a similar segregation of the role of new neurons according to their birth place (posterior/dorsal versus anterior/ventral dentate gyrus) (for reviews see Fanselow and Dong, 2010; Moser and Moser, 1998; Strange et al., 2014).

The hippocampus is not only involved in regulating the stress response, it is also very sensitive to the effects of stress. In particular, two macroscopic and two microscopic categories of hippocampal biomarkers have been shown to be sensitive to stress. The microscopic categories, which are quantified post-mortem, are the rate of neurogenesis, defined as the rate of precursor cell proliferation and/or the rate of new neuron incorporation, and the structural characteristics of mature neuronal cell bodies (cell body size, size/complexity of the dendritic tree, dendritic spine density). Macroscopic biomarkers, which reflect without distinction the two microscopic biomarkers at a larger scale, are the size of the hippocampus (volume of the whole or of the anterior/ventral hippocampus), and the local amount of grey matter in the (anterior/ventral) hippocampus. The local amount of grey matter is typically measured *in-vivo*, using magnetic resonance imaging, while hippocampal volume can be measured *in-vivo* or *ex-vivo*.

## 3 Can we validate hippocampal biomarkers of cumulative affective experiences?

Validation of new markers usually requires the establishment of three different types of validity: 1) criterion validity, which examines the correlation between the new marker and a pre-existing marker considered to be the current gold standard, where such exists; 2) construct validity, which shows whether a marker follows relevant theoretical assumptions of the phenomenon of which it is a marker; and 3) content validity, which refers to the extent to which a marker encompasses all facets of a given construct (Cronbach and Meehl, 1955; Trochim et al., 2015). The distinction between construct and content validity is to some extent artificial. Indeed, if encompassing all the facets of the construct is considered one of the theoretical assumptions the marker should fulfil, content validity becomes a sub-type of construct validity (Trochim et al., 2015). We follow the approach of combining the assessment of construct and content validity.

### 3.1 Criterion validity

Establishing criterion validity requires comparison of the new marker to a pre-existing marker considered as the current gold standard, where such exists. However, there is currently no gold standard method for measuring cumulative affective experience in non-human animals. We therefore turn to data from humans to explore criterion validity.

Two psychological constructs closely related to cumulative affective experience in humans are self-esteem and subjective psychological well-being. Subjective psychological well-being is a self-report measure of life satisfaction driven by autonomy, environmental mastery, personal growth, positive relations with others, purpose in life and self-acceptance (Ryff and Keyes, 1995). Self-esteem is a broadly defined personality variable referring to the degree to which an individual values and accepts him or herself (Pruessner et al., 2005) and is a strong predictor of subjective psychological well-being (e.g. Kubarych et al., 2012). One would thus predict hippocampal biomarkers to correlate with these constructs in humans. In accordance with our predictions, subjective well-being (Van ‘t Ent et al., 2017) and self-esteem (Kubarych et al., 2012) are positively correlated with hippocampal volume. Thus, there is evidence to suggest that hippocampal biomarkers correlate with psychological concepts in humans, which are close to the concept of cumulative affective experience.

Another related concept in humans is mood. Moods are usually considered to be long-lasting affective states resulting from the integration of positive and negative acute experiences over time (Mendl et al., 2010; Nettle and Bateson, 2012). Although the time window in which mood and cumulative affective experience integrate acute experiences could be different (possibly shorter for mood), the construct of mood is very close to that of cumulative affective experience. In humans, moods can be verbally reported and systematically assessed via structured questionnaires. Several meta-analyses have shown that both hippocampal volume and the local amount of grey matter in the hippocampus are consistently lower in patients suffering from two clinically-defined mood disorders: major depression (Arnone et al., 2016, 2012; Koolschijn et al., 2009; McKinnon et al., 2009) and post-traumatic stress disorder (Bromis et al., 2018; O’Doherty et al., 2015; Woon et al., 2010). Longitudinal studies have also shown that various mood-improving treatments (anti-depressant drugs, electro-convulsive therapy) induce an increase in hippocampal volume and the local amount of hippocampal grey matter in depressed patients (Abbott et al., 2014; Arnone et al., 2013; Frodl et al., 2008). Human data therefore show that some of our proposed hippocampal biomarkers co-vary with long-term affective states, as would be required in order to establish criterion validity.

Moods are difficult to assess objectively in non-verbal animals. However, neuroscientists have developed several behavioral tests that are argued to measure mood in laboratory animals (e.g. elevated plus maze; open field test; novelty-suppressed feeding test; anhedonia). These tests have been validated using drugs shown to have clinical efficacy in treating human mood disorders (Christmas and Maxwell, 1970; Dulawa et al., 2004; Merali et al., 2003; Pellow et al., 1985; Pellow and File, 1986; Wallace-Boone et al., 2008). Using such tests, hippocampal volume, local amount of grey matter, and neurogenesis rate have all been shown to be reduced in rodent and macaque models of depression and anxiety, and to be increased by anti-depressant drugs (e.g. Mohammad et al., 2016; Morais et al., 2017; Perera et al., 2011; Santarelli et al., 2003; Snyder et al., 2011; Suzuki et al., 2015; Willard et al., 2009; Wu et al., 2014). Furthermore, increased neurogenesis has been shown to be necessary to observe some of the behavioral effects of anxiolytic and anti-depressant treatments in stressed animals (Mohammad et al., 2016; Perera et al., 2011; Santarelli et al., 2003; Wu and Hen, 2014; Zheng et al., 2017). Although suppression of neurogenesis achieved by transgenic manipulation or irradiation has been shown to be sufficient to induce depressive behavioral symptoms in some studies (e.g. Snyder et al., 2011), in many others it did not result in any changes in depression-like behaviors, unless further stressors were also experienced (reviewed in Miller and Hen, 2015). The most recent hypothesis on the causal role of hippocampal neurogenesis in depression and anxiety symptoms is that low levels of neurogenesis make an animal (and human) more sensitive to environmental stressors (Anacker et al., 2018). It is therefore clear that conditions that lead to changes in mood-like states in animal models of depression and anxiety also change hippocampal biomarkers, and the current thinking is that the hippocampal structures are involved in mediating this mood response, either directly or indirectly.

In summary, there is robust evidence from non-human mammals, and to a lesser extent from humans, for a strong association between changes in mood and hippocampal biomarkers. This evidence demonstrates the criterion validity of hippocampal biomarkers for the assessment of cumulative affective experience.

### 3.2 Construct (and content) validity

Construct validity refers to the extent to which a marker follows theoretical assumptions of the construct it is proposed to reflect. A good marker of cumulative affective experience should fulfil the following assumptions: 1) it should respond to a wide range of events inducing changes in enduring affective states and co-vary in opposite directions with events inducing positively- and negatively-valenced experiences; 2) it should reflect the affective response of each individual to an event, rather than the objective event itself; and 3) it should integrate discrete experiences over time (Bateson, 2016; Bateson and Poirier, 2019).

#### 3.2.1 Assumption 1: co-varying with positive and negative experiences

A large number of studies in different mammalian species (mice, rats, tree shrews, marmosets, macaques and humans) have measured the impact on the hippocampus of events known to induce a negative long-lasting affective state (a search on PubMed with keywords ‘Chronic stress’ ‘Hippocampus’ and ‘Mammals’ returned 3104 items at the date of writing). In humans, meta-analyses have consistently found associations between psychological trauma and a smaller hippocampal volume or local amount of grey matter (Bromis et al., 2018; Paquola et al., 2016; Woon et al., 2010). In non-human species, the vast majority of studies revealed that the macro and microscopic hippocampal biomarkers decrease with chronic exposure to a variety of aversive events such as restraint, social defeat, social isolation and maternal neglect (no meta-analyses seem to have been performed). While most human studies are correlational, experimental animal studies have demonstrated the causal role of chronic stressors in decreased hippocampal volume (e.g. Rahman et al., 2016), local amount of grey matter (e.g. Jackowski et al., 2011), neurogenesis rate (e.g. Lehmann et al., 2013; Lemaire et al., 2000; Mitra et al., 2006; Perera et al., 2011; Pham et al., 2003) and size of cell bodies and dendritic trees (e.g. Magariños et al., 1996; Magariños and McEwen, 1995a).

Cumulative affective experience does not have to be only negative (as had been implied by the previously-used terminology ‘cumulative severity’) (Pickard, 2013). Consequently, to possess content validity (i.e. to include all the facets of the construct), markers of cumulative affective experience must co-vary with exposure to events inducing negative and positive experiences in opposite directions. The case for negative experiences decreasing the hippocampal biomarkers we are reviewing here is strong and incontrovertible (see above). However, crucially, these same hippocampal biomarkers have also repeatedly been found to increase when individuals were chronically exposed to events known to induce positive affective states. Systematic reviews and meta-analyses have shown that voluntary physical activity and mindfulness meditation, which are both associated with positive effects on mood in humans (Brown and Ryan, 2003; Reed and Buck, 2009), increase hippocampal volume in human subjects (Firth et al., 2018; Fox et al., 2014). Results from randomized controlled trials show that exposure to events inducing enduring positive affective states cause the changes in hippocampal biomarkers (Erickson et al., 2011; Holzel et al., 2011; Thomas et al., 2016). In rodents as well, sexual behavior, voluntary physical activity, and cage enrichment, which are well-established rewarding events (Schoenfeld and Gould, 2012), have been experimentally shown to consistently increase rodent hippocampal volume (e.g. Sierakowiak et al., 2015), neurogenesis rate (e.g. Bednarczyk et al., 2009; Eadie et al., 2005; Kempermann et al., 1997; Kodali et al., 2016; Lehmann et al., 2013; Leuner et al., 2010; Snyder et al., 2009; Tanti et al., 2012; van Praag et al., 1999), and the size of the dendritic tree and the spine density of hippocampal neurons (e.g. Eadie et al., 2005; Sierakowiak et al., 2015; Stranahan et al., 2007). In marmosets, cage enrichment has also been shown to enhance the length and the complexity of the dendritic tree of hippocampal neurons (Kozorovitskiy et al., 2005). In rodents and non-human primates, diverse events inducing enduring positive affective states have thus been shown to cause an increase in the different hippocampal biomarkers.

The effect of exposure to aversive events on the hippocampus is known to be mediated, at least partially, by high levels of corticosteroids (Cameron and Gould, 1994; Magariños and McEwen, 1995b; Tanapat et al., 2001). However, chronic exposure to rewarding events (physical and sexual activity, cage enrichment) is also known to be associated with an increased concentration of circulating corticosteroids (Schoenfeld and Gould, 2012) and in one rodent study, corticosteroids were found necessary both for the effect of cage enrichment on neurogenesis enhancement, and for the effect of chronic stress on decreased neurogenesis (Lehmann et al., 2013). These studies indicate that the relationship between corticosteroids and the hippocampal biomarkers is complex and illustrate neatly that measuring a change in corticosteroid levels will not predict the direction of a change in hippocampal biomarkers and affective state valence.

#### 3.2.2 Assumption 2: reflecting individual response, not objective event

The affective reaction of an individual, whether conscious or not, depends not only on the event itself (its nature, its intensity or length) but also on characteristics specific to the individual, including its genotype and its previous experiences, both of which may affect how the individual responds to a given event. Using the number of events known to have the potential to induce a change in affective experience as a proxy for cumulative experience of an individual can thus be inaccurate. A good marker of cumulative affective experience should reflect the impact of events at the individual level; in other words: the individual’s response to the events.

In support of this assumption, hippocampal biomarkers have been shown to depend on the interaction of exposure to aversive or rewarding events with genetic variants in humans (Gatt et al., 2009; Rabl et al., 2014) and mice (Ieraci et al., 2016). This suggests that different individuals respond differently to the same events, and that hippocampal biomarkers reflect the response, rather than the event. A more direct way to test whether a marker reflects the response of each individual is to consider studies in which exposure to an event induced inter-individual differences in the behavioral reaction of the subjects; in such studies, a marker of cumulative affective experience should track these individual differences in response. Accordingly, the neurogenesis rate in the hippocampus of mice (Mitra et al., 2006) was found to negatively correlate with the behavioral manifestation of stress expressed by each individual, despite the fact that all individuals had been experimentally exposed to the same stressful events.

#### 3.2.3 Assumption 3: integrating experiences over time

Depending on their intensity and duration, as well as the genotype and previous experiences of the individual, some affective experiences leave a long-lasting trace and thus have the potential to accumulate over time, while others do not (Bateson, 2016; Bateson and Poirier, 2019). To track cumulative affective experience, hippocampal biomarkers should thus be sensitive to the experiences leaving lasting traces and have the potential to integrate these affective experiences over time, on the same time scale as the affective experiences themselves. Therefore, if the animal completely recovers from an affective experience, and does not experience any lasting effects, we should also not expect a trace in the hippocampal biomarkers. On the other hand, if there are long-lasting changes in affect, then there should also be long-lasting changes in hippocampal biomarkers. Several studies in humans and rodents have shown that effects of affective experiences on hippocampal biomarkers can be long lasting.

One set of examples where the long-lasting effect of affective experiences is very clear is for stressors experienced during development. For instance, effects of early life experiences on hippocampal biomarkers have been detected in adult rats (Lemaire et al., 2000), macaques (Jackowski et al., 2011) and humans (Dannlowski et al., 2012). It is worth noting that lab and farm animals are rarely given the opportunity to live beyond adolescence/young adulthood. In these instances, hippocampal biomarkers will very likely track their cumulative experience over their entire (short) lifetime.

Some could argue that stressful events experienced during development have qualitatively different effects on the brain than those experienced during adulthood. In order for hippocampal biomarkers to be useful markers of cumulative affective experience in any situation/independently of the age of the subject, they have to integrate events encountered during adulthood as well. This cumulative property of hippocampal biomarkers is supported by several studies showing that they correlate with the duration or number of discrete acute experiences. For example, in adult rats and mice, neurogenesis increases with the duration animals spend voluntarily running in a wheel over 4–8 weeks (Bednarczyk et al., 2009; Kodali et al., 2016). The cumulative nature of the hippocampal biomarkers is also confirmed by longitudinal data demonstrating dose effects within subjects. For instance, the local amount of grey matter in the hippocampus decreases with the number of stressful events adult human participants experienced over the last three months (Papagni et al., 2011); the volume of the hippocampus increases with the duration (tested up to 12 months) of the physical exercise program older human participants were enrolled in (Erickson et al., 2011); and the whole hippocampal volume decreases proportionally to the number of days or weeks rats have been exposed to a stressful paradigm (Luo et al., 2014; Rahman et al., 2016).

To integrate positive and negative experiences over time, markers should not just be long-lasting, but positive and negative experiences should also be able to (at least partially) cancel each other out. Other experimental studies in animal models have indeed shown that the hippocampal biomarkers reflect the net effect of combinations of events that provoke affective states of opposite valence within the same individuals (Kim et al., 2013; Li et al., 2017; Morais et al., 2017). It should therefore definitely be possible to assess cumulative affective experience if we restrict ourselves to measuring changes in hippocampal biomarkers over short periods (a few months). When changes are measured over longer periods, we should be aware that an absence of significant differences might potentially be attributable to the potential lack of sensitivity of the method to experiences that happened during adulthood but a long time (years) ago.

Therefore, these studies indicate that the ability of the hippocampal biomarkers to integrate discrete affective experiences over time does not seem to be restricted to a specific period of life. Hippocampal biomarkers have been found to be sensitive to the accumulation of discrete experiences occurring during childhood (Dannlowski et al., 2012; Hodel et al., 2015) as well as during early (Bednarczyk et al., 2009; Li et al., 2017; Luo et al., 2014; Papagni et al., 2011; Rahman et al., 2016) and late adulthood (Erickson et al., 2011). The exact length of the integration window needs to be studied in more detail, but it is clear that in some instances, it can be very long: the hippocampal volume of human subjects has been found to correlate with the number of major stressful events they experienced over their whole life (Rabl et al., 2014).

### 3.3 Potential confounding variables

We have established that hippocampal biomarkers are closely associated with the enduring affective states of mammals, and may even be involved in modulating these affective states. However, processes other than affective states can also influence these hippocampal biomarkers. To be able to interpret a hippocampal biomarker in terms of cumulative affective experience it is therefore necessary to control for these potential confounding variables. Hippocampal biomarkers are known to vary for various reasons unrelated to affective state including age, sex, total brain size (for hippocampal volume and amount of grey matter only), genotype, and the non-affective component of experiences (learning and memory) (e.g. Fjell et al., 2013; Maguire et al., 2000; Schoenfeld and Gould, 2012; Strange et al., 2014; Sullivan et al., 2001; Walhovd et al., 2011). Some of the microscopic hippocampal biomarkers are also known to change with the acute affective state of an individual (Leuner and Shors, 2013; Schoenfeld and Gould, 2012; Strange et al., 2014). These various potential confounding factors need to be taken into account when using hippocampal biomarkers to assess cumulative affective experience.

#### 3.3.1 Age, sex, total brain volume and genotype

It is important to be aware of these confounding variables, but generally, they can be eliminated by good experimental design and/or statistical analysis. For instance, one can choose to study individuals of the same age or same sex. Age and sex can also be matched between groups that are to be compared. Genotype can be controlled for by comparing large groups of individuals (assuming that each group is representative of the whole population, genetic variation should be balanced between groups). For markers that can be quantified *in vivo*, the effect of genotype and sex can also be eliminated by using a longitudinal design and studying within-subject effects. Factors including age, sex and total brain volume can also be controlled for by including them as covariates in the statistical analyses (in this case, it is necessary to establish that control variables are not strongly correlated with the predictor variable of interest).

#### 3.3.2 Learning and memory processes

As described previously, the hippocampus is anatomically and functionally divided along its longitudinal axis, with the anterior/ventral part being more involved in affective states and the posterior/dorsal part more involved in learning and memory (for reviews see Fanselow and Dong, 2010; Moser and Moser, 1998; Strange et al., 2014). This functional segregation does not seem to be limited to acute affective states but extends to enduring ones. For instance, a recent study showed that granule cell activity in the anterior/ventral dentate gyrus has anxiogenic effects, and that new neurons in the anterior/ventral dentate gyrus inhibit this activity, conveying more stress resilience to the animals (Anacker et al., 2018). Even if this spatial segregation is not absolute and some counter-examples have been described (Tanti and Belzung, 2013), it should be possible to reduce the probability that non-affective changes affected hippocampal biomarkers by focusing specifically on the anterior/ventral hippocampus. Measures focusing on the anterior/ventral part of the hippocampus should be more sensitive than those applied to the whole structure. Indeed, an increase in a hippocampal biomarker in the anterior/ventral part can sometimes be accompanied by a decrease in the posterior/dorsal parts of the hippocampus (Maguire et al., 2000); in such circumstances, a whole hippocampal volume approach is likely to lead to false-negative results.

A complementary approach for excluding non-affective confounders consists of combining the hippocampal biomarker with another marker of affective state. For instance, cumulative measurement of corticosteroids (for instance in hair) could be used. Although corticosteroid levels are believed to be more sensitive to arousal than affective valence (Otovic and Hutchinson, 2015; Paul et al., 2005; Ralph and Tilbrook, 2016), a change in valence is usually associated with a change in arousal, independently of the direction of the change in arousal: mood-deteriorating chronic stress, and mood-improving physical activity, sexual behavior and cage enrichment are usually associated with an increase in corticosteroid levels, whereas mood-improving mindfulness meditation (low arousal) is associated with a decrease (see Section 3.2.1). In contrast, corticosteroid levels are not expected to change due to learning and memory processes alone (when not associated with a change in affective processes). Therefore, if a change in corticosterone levels is found to accompany a change in a hippocampal biomarker this should rule out the hypothesis that the latter change is only due to a change in non-affective cognitive processes.

#### 3.3.3 Acute affective states

Acute stressors can also decrease neurogenesis (Schoenfeld and Gould, 2012) and alter the structural characteristics of mature neuronal cell bodies (Leuner and Shors, 2013). The microscopic hippocampal biomarkers can only be taken post-mortem and thus require prior euthanasia. Even when the animal is killed in the most humane way possible, the event might still induce some acute stress and thus potentially impact the hippocampal biomarkers. Comparing groups exposed to the same euthanasia protocol should control for this potential confounding factor. In the specific case of neurogenesis, quantifying markers of late stages of neural differentiation (which are unaffected by of what happens in the last 24 h, Schoenfeld and Gould, 2012) should also eliminate this potential confound.

Macroscopic neuroimaging *in-vivo* hippocampal biomarkers (volume, local amount of grey matter) require anesthesia or head restraint, two procedures that could potentially induce some acute stress. So far, macroscopic hippocampal changes have never been observed after acute stress, probably because the scale of the changes occurring after acute stress are too small to be detected with this approach. However, with technical improvement, it might become a problem for longitudinal designs in the future, or even today if measurements are taken at a high frequency (for instance daily), in which case the stress could become chronic. In this case, the number of measurements should be included in the statistical models to control for potential stress induced by the measurements themselves.

### 3.4 Interim conclusion

Hippocampal biomarkers are closely associated with enduring affective states in various species of mammals. They co-vary with a wide range of events inducing positive and negative affective states. There is some evidence to suggest that hippocampal biomarkers track the enduring affective states of mammals taking into account their individual responses, rather than the events. Hippocampal biomarkers have been shown to reflect the accumulation of positive and negative affective experiences over long periods of time and possibly the whole life of individuals. This evidence is strong enough to start using these biomarkers as indicators of cumulative affective experience in the context of animal welfare. Only wider use of such biomarkers will allow us to determine the limits of their usefulness as welfare indicators.

## 4 The hippocampus as a biomarker of cumulative affective experience in non-mammalian vertebrates

The hippocampus is an evolutionarily conserved region, and homologues have been described in all vertebrate lineages (Bingman et al., 2009). The stress response system, including the HPA axis is also highly conserved in vertebrates (Denver, 2009). This opens the possibility that the hippocampal biomarkers described above might also be an indicator of cumulative affective experience in non-mammalian vertebrate species.

In birds, adult neurogenesis takes place in numerous brain regions throughout life, including the hippocampus. The role of the avian hippocampus in (especially spatial) learning and memory is well established (Roth et al., 2010) and new-born neurons are suspected to play an important role in this process (LaDage, 2016). A potential role of the hippocampus and its new-born neurons in emotional regulation is only starting to emerge (Smulders, 2017). As in mammals, the avian hippocampus has high expression levels of glucocorticoid receptors and the density of these receptors, especially mineralocorticoid receptors, is regulated by stress (Banerjee et al., 2012; Dickens et al., 2009; Zimmer and Spencer, 2014). Several studies have shown a reduction of avian hippocampal neurogenesis and/or volume in potentially stressful situations (LaDage et al., 2010; Nikolakopoulou et al., 2006; Pravosudov and Omanska, 2005; Tarr et al., 2009; Taufique et al., 2018) and an increase with enrichment (LaDage et al., 2010; Melleu et al., 2016). However, in all these cases, the authors have either favored or been unable to exclude the possibility that the effects were due to a change in spatial memory abilities (see also Roth et al., 2012). Recently, food restriction was found to be associated with reduced hippocampal neurogenesis (but not total volume) and chronically elevated corticosterone levels in chickens (Robertson et al., 2017). In this paradigm, changes are unlikely to be driven by a change in spatial memory. Nevertheless, there is a clear need for more controlled experiments testing the specific role of positive and negative enduring affective states on the avian hippocampus volume and neurogenesis.

In fish, neurogenesis takes place in numerous parts of the brain, one of them being homologous to the mammalian hippocampus (the lateral area of the dorsal telencephalon) (Zupanc, 2008). Due to this ubiquity, neurogenesis has usually been assessed in the whole brain, without differentiating the results according to brain regions. As in mammals, brain neurogenesis in fish seems to respond in opposite directions to positive and negative affective experiences. Down-regulation of neurogenesis by stress has been shown in various species of fish (electric fish, rainbow trout, cichlid), using different stressors (predation pressure, tail injury, social stress) (Dunlap et al., 2016; Maruska et al., 2012; Sørensen et al., 2012; Tea et al., 2018). In contrast, environmental enrichment was found to increase brain cell proliferation in electric fish and zebrafish (Dunlap et al., 2011; von Krogh et al., 2010). Dose-dependency has also been found, with cell proliferation correlating with the predation or social pressure (Dunlap et al., 2016; Sørensen et al., 2012). However, teleost fish have a very high rate of adult neurogenesis compared to mammals (Zupanc, 2008), and whether a neurogenesis marker has the capacity to integrate (positive and negative) experiences over long periods of time is currently unknown.

## 5 Implementation of hippocampal biomarkers

We currently cannot interpret absolute values of any hippocampal biomarker, because we lack quantitative definitions of what constitutes good or bad cumulative affective experience. Consequently, only relative measures (changes over time or differences between groups or individuals) can be interpreted. This problem is common to any welfare indicator. The main potential of these hippocampal biomarkers we envisage for the near future is thus to compare the effects of different housing conditions or husbandry and experimental procedures on the cumulative experience of animals, rather than assessing the absolute cumulative affective experience of individual subjects.

Macroscopic hippocampal biomarkers can be measured *in-vivo*, allowing repeated measures on the same animals. *In-vivo* measurements require access to magnetic resonance imaging facilities with strong magnets. While such equipment is progressively becoming widespread in academic and industrial biomedical settings, it is usually not available in farm settings (except on a few experimental farms). Macroscopic markers thus have the potential to be used as a research tool, but not as a practical technique for on-site welfare assessment.

Microscopic hippocampal biomarkers need to be taken post-mortem; consequently, only between-subject designs are possible. However, the microscopic biomarkers do not require expensive equipment in proximity to the animals, as brains can be collected when animals are slaughtered and processed elsewhere. Their application field thus seems wider compared to macroscopic biomarkers, although still limited.

Hippocampal biomarkers do not require much time with the (live) animal compared with existing behavioral markers of mood (none for the post-mortem microscopic biomarkers, a couple of hours for the *in-vivo* macroscopic biomarkers), which can be convenient when access to the animals is time limited. They do, however, require intensive data processing and technical skills. On-going studies are trying to validate innovative ways to assess neurogenesis rate by quantifying messenger RNA of genes involved in the process (for instance doublecortin) using quantitative PCR rather than immuno-histochemical methods to detect proteins (Gualtieri et al., 2017). Such a technique would speed up the data processing and should facilitate the implementation of this approach. Meanwhile, we envisage hippocampal biomarkers being used by a small number of experts who can provide information useful for a large number of stakeholders, for instance by comparing the welfare impact of different protocols involving multiple events.

The validation of hippocampal biomarkers of cumulative affective experience mainly relies on data from human and non-human primates and from rodents. Considering how conserved the biology of the hippocampus and the HPA axis is among mammals, however, we expect these biomarkers to be valid in any mammalian species. The macroscopic biomarkers (hippocampal volume and local amount of grey matter) depend on two main microscopic underlying mechanisms, neurogenesis and structural plasticity of mature neuronal cell bodies. It is possible that the respective contribution of the two microscopic mechanisms varies between mammalian species (Sorrells et al., 2018; Moreno-Jiménez et al., 2019, but see Boldrini et al., 2018 and Kempermann et al., 2018). Since the two microscopic biomarkers seem to have the same properties, this variation does not matter when one uses the macroscopic biomarkers. However, we would advise any researcher interested in using one of the microscopic biomarkers in a new species to first verify that their chosen marker is sensitive to a validated manipulation of affective state.

## 6 Conclusion

This review of recent findings in stress biology and psychiatry suggests that various mammalian structural hippocampal biomarkers have criterion, construct and content validity for assessing the cumulative affective experience of individuals. These hippocampal biomarkers seem to offer a promising objective method to identify which husbandry conditions or experimental procedures induce a deterioration or amelioration of the cumulative affective experience of captive mammals, and to test the efficacy of any attempted refinements. The *in-vivo* biomarkers also potentially provide an opportunity to better define humane end-points, hence decreasing potential animal suffering. *In-vivo* biomarkers could also play a role in assessing the quality of life of humans unable to self-report their well-being (e.g. stroke, coma). More data are required to validate the *in-vivo* and *ex-vivo* biomarkers in non-mammalian species. We hope that this analysis will motivate welfare researchers, neuroscientists and clinicians to explore the potential of these new biomarkers.
